# The Impact of the Definitions of Clinical Phases on the Profiles of Grey-Zone Patients with Chronic Hepatitis B Virus Infection

**DOI:** 10.3390/v15051212

**Published:** 2023-05-22

**Authors:** Xiaoqian Xu, Hao Wang, Shan Shan, Yameng Sun, Xiaoyuan Xu, Hong You, Jidong Jia, Hui Zhuang, Yuanyuan Kong, on behalf of the China Registry of Hepatitis B (CR-HepB) Group

**Affiliations:** 1 Clinical Epidemiology and EBM Unit, Beijing Friendship Hospital, Capital Medical University, Beijing Clinical Research Institute, Beijing 100050, China; 2 Liver Research Center, Beijing Friendship Hospital, Capital Medical University, National Clinical Research Center for Digestive Diseases, Beijing 100050, China; 3 Department of Infectious Diseases, Peking University First Hospital, Beijing 100034, China; 4 Department of Microbiology and Infectious Disease Center, Peking University Health Science Center, Beijing 100191, China

**Keywords:** chronic hepatitis B, antiviral therapy, grey zone, indeterminate phase

## Abstract

We aim to investigate the impact of different clinical phases’ definitions of chronic hepatitis B (CHB) infection on the profiles of grey zone, based on HBV guidelines set by the Chinese Society of Hepatology and Chinese Society of Infectious Diseases (CSH/CSID, 2022 version) and guidelines set by the American Association for the Study of Liver Diseases (AASLD, 2018 version). We retrospectively examined untreated CHB patients enrolled in the China Registry of Hepatitis B database. Patients’ clinical phases were determined as per CSH/CSID and AASLD. Liver fibrosis was estimated by FIB-4 and/or APRI. Among 3462 CHB patients, 56.9% and 41.7% fell into the grey zone based on AASLD and CSH/CSID. Compared with grey zone patients as per AASLD, those under CSH/CSID guidelines showed lower levels of median ALT (26.0 vs. 37.0 U/L, *p* < 0.001), AST (25.0 vs. 29.4 U/L, *p <* 0.001) and APRI (0.3 vs. 0.4, *p* < 0.001), and lower rates of advanced fibrosis estimated by APRI (7.9% vs. 11.4% *p =* 0.001), but comparable rates by FIB-4 (13.0% vs. 14.1%, *p* = 0.389). With the stepwise lowering of ALT upper limits of normal (ULN) values from 50/40 U/L for males/females to 40/40 U/L, 35/25 U/L and 30/19 U/L, the proportions of grey zone patients as per CSH/CSID declined from 46.7% to 41.7%, 34.3% and 28.8%, respectively, whereas they remained stable (55.7%, 56.2%, 56.9% and 57.0%) as per AASLD. Compared with the AASLD guidelines, CSH/CSID guidelines leave fewer and less severe patients in the grey zone. Lowering ALT ULN values reduces the number of grey zone patients as per CSH/CSID, but not under AASLD guidelines.

## 1. Introduction

Chronic hepatitis B virus (HBV) infection follows a dynamic natural course reflecting the interaction between HBV replication and the host immune response. Based on the patient’s virological and biochemical profiles, the clinical course is schematically classified into four phases before HBsAg clearance [1,2,3]. However, many patients do not fit well into these well-described phases, and fall into the grey zone, namely, the “indeterminate phase”. These patients’ characteristics are more mixed, and their clinical significance and optimal management are uncertain [4].

Furthermore, the thresholds of HBV DNA and alanine aminotransferase (ALT) used in different guidelines result in different profiles of patients in the grey zone. Chinese guidelines for the prevention and treatment of chronic hepatitis B (CHB) have adopted ALT ≥ 1 × upper limit of normal (ULN) in defining the phases of chronic hepatitis since 2019, while ALT ≥ 2 × ULN has also been adopted in other guidelines [1]. Furthermore, in the guidelines recently released by the Chinese Society of Hepatology and the Chinese Society of Infectious Disease (CSH/CSID 2022), the thresholds of HBV DNA for defining clinical phases have been extensively revised [5,6]. In addition, there is an emerging trend of lowering the specific treatment threshold for ALT values when deciding to initiate antiviral therapy [1,2,3,7]. However, evidence of how the grey zone would change with varying definitions is limited.

Therefore, in the current study, we used a nationwide registration database for CHB patients in China to investigate the impacts of different definitions of clinical phases in the HBV guidelines set by the CSH/CSID 2022 and the American Association for the Study of Liver Diseases (AASLD 2018) [1] on the profiles of patients in the grey zone, and the impacts of varying ALT ULN on the proportions of grey zone patients.

## 2. Methods

### 2.1. Patient Enrollment

Patient data were extracted from the China Registry of Hepatitis B (CR-HepB), which is a nationwide, web-based electronic platform established in 2012 based on 55 hospitals in China, enrolling patients with chronic HBV infection, namely, with hepatitis B surface antigen (HBsAg) ≥ six months, regardless of treatment status. The demographic and laboratory results were inputted by investigators from electronic medical records. Participants received a standard of care established by the updated national and/or international guidelines. Detailed information on CR-HepB can be found in our previous reports [8,9]. The study was conducted according to the guidelines of the Declaration of Helsinki, and approved by the ethics committee of Beijing Friendship Hospital, Capital Medical University; informed consents were waived.

In the present study, data of untreated CHB patients from July 2012 until October 2022 without clinical diagnoses of cirrhosis (compensated or decompensated), hepatocellular carcinoma (HCC) or liver transplantation were extracted from CR-HepB. Patients were included in the final analysis if they had complete records on hepatitis B e antigen (HBeAg), DNA viral loads, ALT values, and other indicators used for the calculation of the fibrosis score based on four factors (FIB-4) and aspartate aminotransferase-to-platelet ratio index (APRI) index.

### 2.2. Classification of the Patients into Different Phases of Chronic HBV Infection

Patients were classified into the proper phases if they met the corresponding criteria on HBeAg, HBV DNA, and ALT values as per HBV guidelines set by the CSH/CSID 2022 [5] and the AASLD 2018 [1] (Table 1). Given that different terminology relating to clinical phases was adopted in these guidelines, we used phase 1, phase 2, phase 3 and phase 4 in the current study to represent the phases of HBeAg-positive chronic HBV infection (immune tolerant), HBeAg-positive chronic hepatitis B (HBeAg-positive immune active), HBeAg-negative chronic HBV infection (inactive), and HBeAg-negative chronic hepatitis B (HBeAg-negative immune active), respectively. Patients who did not meet the above criteria and fell outside the clearly defined phases were classified as grey zone (indeterminate phase) per each guideline. Since no specific ALT ULN values were recommended in the CSH/CSID 2022, we used the conventional 40 U/L for both males and females in the present analysis.

### 2.3. Characterization of the Grey-Zone Patients

The demographic and laboratory characteristics of grey zone patients have been described and compared between different guidelines. Liver fibrosis burden was estimated with the non-invasive fibrosis score based on FIB-4 and/or APRI. The calculating formula was: FIB-4 = (age × aspartate aminotransferase [AST])/(PLT count × ALT^1/2^) [10]; APRI = [AST/ULN/platelet [PLT] count] × 100 [11]. The presence of advanced fibrosis was defined as FIB-4 ≥ 3.25 and APRI ≥ 1.5, respectively, according to previous studies [10,11].

### 2.4. Impact of Different ALT ULN Values on the Proportion of Patients in the Grey Zone

To investigate the impact of the stepwise lowering of ALT ULN values on the proportion of the patients in the grey zone, four commonly proposed values per each guideline were used: the proposed industry standard of ALT ULN criteria of 50 U/L for males and 40 U/L for females (50/40) in China [12]; the conventional ULN criteria of 40 U/L for both males and females (40/40) adopted by the European Association for the Study of the Liver (EASL) [2] and the Asian Pacific Association for the Study of the Liver (APASL) [3]; the ULN criteria of 35 U/L for males and 25 U/L for females (35/25) as used in the current AASLD guidelines [1], and the ULN criteria of 30 U/L for males and 19 U/L for females (30/19) recommended by expert consensus and in the guidelines set by AASLD (2016 version), NICE, East Asia and CSH [13,14,15,16].

### 2.5. Statistical Analysis

The continuous variables have been described as the median and interquartile range (IQR), and differences between groups were assessed with the Kruskal–Wallis test, considering that most variables did not meet assumptions of normality. Categoric variables were described as numbers and proportions and compared with the chi-squared test. To visualize the shift in clinical phases across different definitions in the guidelines, a Sankey diagram was utilized. Nodes of different colors denote different clinical phases under corresponding guidelines, while ribbon widths indicate the changes in numbers of patients between two nodes.

Statistical analysis was conducted with R version 4.3.6 (Packages “networkD3”, “ggplot2”, “RcmdrMisc”), and a 2-tailed *p* value less than 0.05 was considered statistically significant.

## 3. Results

### 3.1. Patient Characteristics

Of the 16,938 untreated CHB patients without cirrhosis (compensated or decompensated), HCC, or liver transplantation, 13,327 were excluded due to missing values for variables critical to defining phases or to the calculation of FIB-4. An additional 149 patients were excluded due to age (<18 years old). Finally, 3462 CHB patients were included in this study (Supplementary Figure S1). The median age of the patients was 40.6 (31.3, 50.5) years old, with 64.2% being males and 52.1% being HBeAg-positive. The median ALT level was 39.0 (24.0, 78.0) U/L, and median HBV DNA viral loads were 4.5 (3.0, 6.8) log_10_ IU/mL (Supplementary Table S1). In total, 15.1% and 17.1% of the patients had advanced fibrosis, as assessed by FIB-4 and APRI, respectively (Supplementary Table S1).

### 3.2. Proportion of the Patients in the Grey Zone as Per the AASLD 2018 and the CSH/CSID 2022

**Figure 1:**
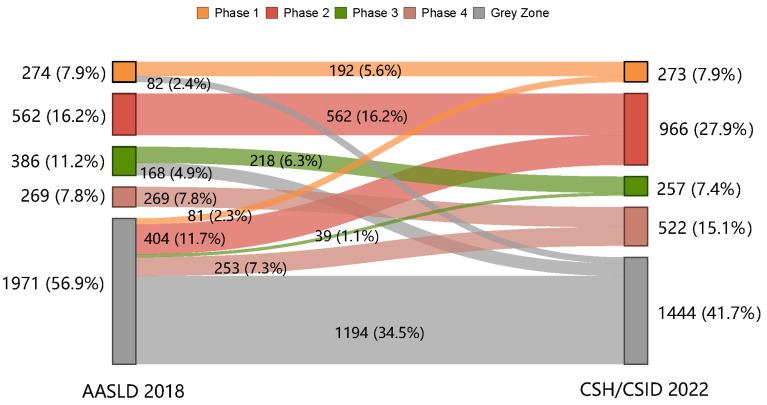
Shift in clinical phases from the AASLD 2018 to the CSH/CSID 2022.

The proportions of the patients in the grey zone were 56.9% and 41.7%, as per the AASLD 2018 and the CSH/CSID 2022, respectively (Figure 1). Besides this, more patients were classified into immune active phases as per the CSH/CSID 2022 (27.9% in phase 2 and 15.1% in phase 4) compared with the AASLD 2018 (16.2% in phase 2 and 7.8% in phase 4).

The shift in clinical phases as per the AASLD 2018 to those as per the CSH/CSID 2022 is shown in Figure 1. A total of 1194 (34.5%) CHB patients fell into the grey zone according to both guidelines (Figure 1). However, another 250 (7.3%) of the patients, including 82 (2.4%) in phase 1 and 168 (4.9%) in phase 3 as per the AASLD 2018, shifted into the grey zone under the CSH/CSID 2022. In contrast, more patients (777, 22.4%) in the grey zone as per the AASLD 2018 were re-classified into explicit phases according to the CSH/CSID 2022 (2.3%, 11.7%, 1.1%, and 7.3% to phases 1, 2, 3, and 4, respectively).

### 3.3. Characteristics of the Grey Zone Patients as Per the AASLD 2018 and the CSH/CSID 2022

The demographic and clinical characteristics of the grey zone patients significantly differed between the AASLD 2018 and the CSH/CSID 2022 guidelines (Table 2). Compared with those as per the AASLD 2018, the grey zone patients as per the CSH/CSID 2022 were slightly older (median age of 44.2 vs. 42.0 years, *p* = 0.001), but exhibited a lower male proportion (57.8% vs. 64.3%, *p <* 0.001), HBeAg-positive rate (39.0% vs. 49.0%, *p <* 0.001), and median levels of ALT (26.0 vs. 37.0 U/L, *p <* 0.001), AST (25.0 vs. 29.4 U/L, *p <* 0.001) and APRI score (0.3 vs. 0.4, *p* < 0.001). The presence of advanced fibrosis was lower under the CSH/CSID 2022 than under the AASLD 2018, as estimated by APRI (7.9% vs. 11.4%, *p =* 0.001), but was similar between the two guidelines as estimated by FIB-4 (13.0% vs. 14.1%, *p* value *=* 0.389).

Further comparisons of inconsistent grey zone patients defined by the two guidelines show that the grey zone patients defined according to the CSH/CSID 2022 alone had a lower proportion of advanced fibrosis estimated by both APRI and FIB-4 (APRI ≥ 1.5: 0.8% vs. 14.4%, *p <* 0.001; FIB-4 ≥ 3.25: 6.8% vs. 13.8%, *p* = 0.005) compared to those defined by the AASLD 2018 alone. In addition, the median levels of ALT (21.0 vs. 48.0 U/L, *p* < 0.001), AST (21.6 vs. 34.0 U/L, *p* < 0.001), and HBV DNA (3.0 vs. 4.9 log_10_ IU/mL, *p* < 0.001) were also lower in these patients (Supplementary Table S2). Specifically, in HBeAg-negative patients with HBV DNA detectable at 2000 IU/mL, the proportions of APRI ≥ 1.5 and FIB-4 ≥ 3.25 were 1.5% and 5.8% for patients with ALT<1 × ULN, and 14.0% and 16.0% for patients with ALT of 1-2 × ULN, respectively.

### 3.4. Changes in the Proportions in Grey Zone Patients with Varying ALT ULN Values as Per the AASLD 2018 and the CSH/CSID 2022

**Figure 2:**
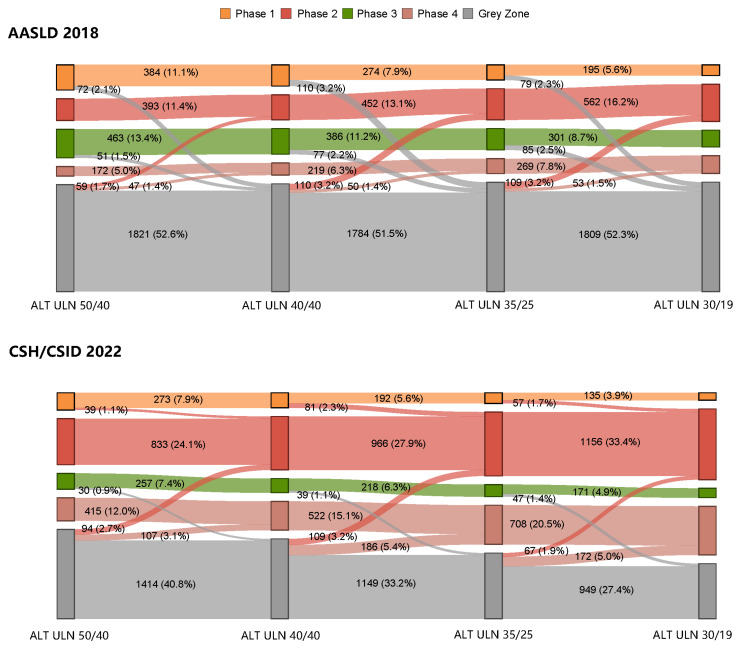
Shift in clinical phases when varying the ALT ULN cutoffs as per the AASLD 2018 and the CSH/CSID 2022.

The number of patients falling into the grey zone as per the CSH/CSID 2022 dramatically declined with the lowering of the ALT ULN values (ALT 50/40: 46.7%, ALT 40/40: 41.7%, ALT 35/25: 34.3%, and ALT 30/19: 28.8%; Figure 2, Supplementary Table S3). However, when using the AASLD 2018, the proportion of grey zone patients remained relatively stable (ALT 50/40: 55.7%, ALT 40/40: 56.2%, ALT 35/25: 56.9%, and ALT 30/19: 57.0%; Figure 2, Supplementary Table S3).

With the lowering of the ALT ULN values, some of the grey zone patients gradually shifted into two immune active phases, as defined by each guideline (Figure 2). This led to stepwise increases in the number of patients classified into immune active phases via ALT ULN values of 50/40, 40/40, 35/25, and 30/19 (the CSH/CSID 2022: 36.1%, 43.0%, 53.9% and 62.4%; the AASLD 2018: 16.4%, 19.4%, 24.0% and 28.7%; Supplementary Table S3). Furthermore, with the lowering of ALT ULN values, fewer patients from phase 3 shifted into the grey zone according to CSH/CSID 2022 (0.9%, 1.1% and 1.4%) than according to AASLD 2018 (1.5%, 2.2% and 2.5%). Additionally, 2.1%, 3.2% and 2.3% of patients shifted from phase 1 into the grey zone according to the AASLD 2018 while no patients shifted from phase 1 into the grey zone when using CSH/CSID 2022.

### 3.5. Subgroup Analyses by Age and Sex

**Figure 3:**
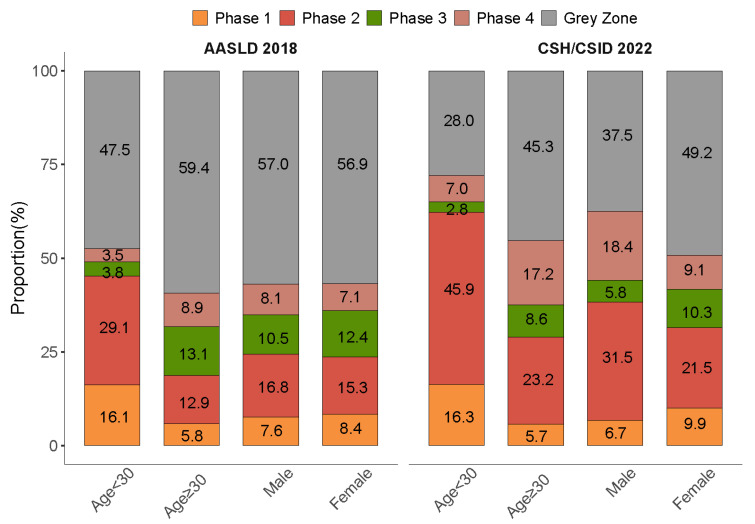
Distribution of clinical phases stratified by age and sex as per the AASLD 2018 and the CSH/CSID 2022.

Subgroup analyses by age showed that around half of the patients fit into the grey zone in the age group ≥ 30 years as per the AASLD 2018 (59.4%) and the CSH/CSID 2022 (45.3%) (Figure 3). In addition, higher proportions of advanced fibrosis evaluated by FIB-4 and abnormal PLT were found in the grey zone patients of the older age group (Table 3).

Subgroup analysis by sex as per the CSH/CSID 2022 revealed a higher proportion of grey zone in females (49.2% vs. 37.5%), and a similar sex-specific proportion of grey zone was found when using the definition per the AASLD 2018 (56.9% vs. 57.0%). The levels of advanced fibrosis as assessed by FIB-4 and APRI and abnormality rates in ALT and PLT were higher in males as per each guideline (Table 3).

## 4. Discussion

Based on the nationwide real-world CR-HepB registry database, we found that compared with the AASLD 2018, the CSH/CSID 2022 identified a lower proportion of grey zone patients. Grey zone patients defined as per the CSH/CSID 2022 carried less severe liver disease estimated by non-invasive markers than grey zone patients as per the AASLD 2018. Furthermore, lowering ALT ULN values significantly decreased the number of grey zone patients under the CSH/CSID 2022, but not the AASLD 2018.

The proportions of untreated CHB patients falling into the grey zone varied depending on the study population. Most studies have adopted the definitions set out in the AASLD guidelines, generating proportions of grey zone patients ranging from 23% to 55% [17,18,19,20]. Our current study revealed that this discrepancy in grey zone proportion in the same population also occurred when defined by different criteria. The lower number of grey zone patients when using the CSH/CSID 2022 compared with using the AASLD 2018 is a combined effect of the lower threshold set for HBV DNA level and the lower folds of ALT ULN levels adopted by the CSH/CSID 2022. The rationale for decreasing the HBV DNA threshold to detectable levels in the CSH/CSID 2022 has been supported by evidence that even low-level persistent viremia can be associated with liver disease progression, and the maximal suppression of HBV may significantly reduce the risk of HCC development [21,22,23]. For patients with mildly elevated serum ALT (1~2 × ULN) who fall into the grey zone according to the AASLD 2018, regular monitoring and consideration for biopsy are recommended [1]. These patients are shifted to two immune-active phases requiring prompt antiviral therapy according to the CSH/CSID 2022. Generally, the criteria set out in the CSH/CSID 2022 are more relaxed in defining the phases of chronic hepatitis, and more stringent in defining the phases of chronic infection. These revisions are anticipated to improve the rate of timely therapy, thereby expanding the uptake of treatment and promoting earlier interventions for CHB patients in China [24]. It is a concern that currently available treatments require a long or indefinite treatment course in order to avoid viral relapse once antiviral therapy is initiated. Nonetheless, the benefits of earlier treatment in the prevention of liver-related events might outweigh those related to the continued use of antiviral drugs given the suboptimal service of linkage to care and poor adherence to continuous monitoring, especially in China [24,25].

Our study has further demonstrated that the severity of grey zone patients, which closely correlates to patient management, differed depending on the criteria used. When defined as per the CSH/CSID 2022, grey zone patients may carry less severe liver disease. However, patient heterogeneity could still occur. Previous studies have reported significantly different liver injury severity levels and long-term prognoses among grey zone patients not eligible for antiviral treatment under the same AASLD criteria [26,27]. Relying solely on clinical phases when managing CHB patients leads to blind spots, and using specific indications proposed in the international or country-specific guidelines [1,2,3] for initiating treatment would be more appropriate. Indeed, it has been recommended that older patients, as well as those with a family history of HCC or cirrhosis, and extrahepatic manifestations, should be considered as showing therapeutic indications [1,2,3,5,6]. Several studies have also identified risk factors for significant liver fibrosis, such as high–normal ALT at 20~40 U/L and HBV DNA ≥ 2000 IU/mL [26], as well as risk factors predicting HCC, specifically in grey zone patients [28]. These factors, together with novel markers, might help us to identify high-risk patients who need prompt antiviral therapy, even if they are in the grey zone [4].

Since grey zone patients are an ill-defined population, minimizing the number of grey zone patients would be desirable. Through the stepwise lowering of ALT ULN values, which are not uniform across countries and/or regions [29], the number of grey zone patients was significantly reduced from 46.7% to 28.8% based on the CSH/CSID 2022, as expected, but it remained stable at around 56% according to the AASLD 2018. It could be speculated that in certain guidelines [2] and simplified treatment strategies [24,30,31] that propose > 1 × ULN as the threshold defining immune active phases, the number of patients in the grey zone would significantly decline with reductions in ULN values. Lowering the ALT threshold would also facilitate screening for significant liver injuries, thereby expanding treatment eligibility [32,33]. It is worth noting that even when the threshold of HBV DNA levels is minimized, as in the CSH/CSID 2022 and when ALT ULN was lowered to 30/19 U/L, the grey zone is still not eliminated. Therefore, developing a strategy to optimize the management of grey zone patients remains a demanding task. The future development and validation of novel biomarkers that better reflect intrahepatic cccDNA and viral replication, ideally combined with demographic and histological factors, might help to further characterize clinical phases and enhance management decisions [29,34].

The most pressing challenge in China is to manage the vast “reservoir” of CHB patients and provide timely antiviral treatments to prevent disease progression [35]. The currently used clinical first-line NA drugs have high efficiency, low drug resistance, and a low incidence of side effects. Specifically, the National Centralized Drug Procurement (NCDP) policy in China has significantly reduced drug costs, making antiviral treatment more accessible to patients [36]. Therefore, an appropriate updating of the CSH/CSID 2022 would contribute to expanding antiviral treatment for CHB patients in China. Although specific ALT ULN values have not been explicitly recommended in the CSH/CSID 2022, the stepwise lowering of ALT ULN values would help to further minimize the number of patients in the grey zone, and increase the uptake of antiviral therapy.

Our study has several limitations. Firstly, due to the limited availability of histology results, non-invasive indicators were used to evaluate the severity of fibrosis in our study. Secondly, only a cross-sectional evaluation was conducted. Longitudinal studies would more effectively address this issue. Nevertheless, a cross-sectional evaluation could still help to outline the phase distribution, and would allow for relative comparisons between guidelines. Besides this, the development of simplified criteria and the prompt initiation of antiviral treatment are also commonly being suggested to help overcome the barriers to HBV elimination [24,30,31]. Lastly, this study did not consider the quantitative measurement of HBsAg, which is used for clinical phase classification in the CSH/CSID 2022. This might have led to an underestimation of the number of grey zone patients.

## 5. Conclusions

In summary, the CSH/CSID 2022 definitions yield lower proportions of grey zone patients, with less severity, than the AASLD 2018 definitions. A decrease in the grey zone population would be beneficial to treatment expansion. However, it will be hard to eliminate the grey zone even after stepwise optimization via ALT ULN. Therefore, the development and validation of novel and specific biomarkers that could accurately classify clinical phases of CHB is justified.

## Figures and Tables

**Table 1:** Phases of chronic HBV infection defined by the AASLD 2018 and the CSH/CSID 2022.

	Phase 1	Phase 2	Phase 3	Phase 4	Grey Zone
Terminology					
CSH/CSID 2022	HBeAg-positive chronic HBV infection	HBeAg-positive chronic hepatitis B	HBeAg-negative chronic HBV infection	HBeAg-negative chronic hepatitis B	Grey Zone/Indeterminate phase
AASLD 2018	Immune tolerant	HBeAg-positive immune active	Inactive	HBeAg-negative immune active	
**HBsAg**					
CSH/CSID 2022	>1 × 10^4 a^	Detectable	<1 × 10^3 a^	Detectable	
AASLD 2018	Present for ≥ 6 months				
**HBeAg**	Positive	Positive	Negative	Negative	Patients who do not fit into any of the criteria of phases 1~4 were deemed grey zone.
**HBV DNA, IU/mL**					
CSH/CSID 2022	>2 × 10^7^	Detectable	Undetectable	Detectable	
AASLD 2018	>10^6^	>2 × 10^4^	<2 × 10^3^	≥2 × 10^3^	
**ALT, U/L**					
CSH/CSID 2022 ^b^	<1 × ULN	≥1 × ULN	<1 × ULN	≥1 × ULN	
AASLD 2018 ^c^	<1 × ULN	≥2 × ULN	<1 × ULN	≥2 × ULN	

Abbreviations: AASLD, American Association for the Study of Liver Diseases; ALT, alanine aminotransferase; CSH/CSID, Chinese Society of Hepatology/Chinese Society of Infectious Diseases; HBeAg, hepatitis B e antigen; HBsAg, hepatitis B surface antigen; HBV, hepatitis B virus; ULN, upper limit of normal. ^a^ Due to the limited availability of values of HBsAg, quantitative HBsAg were not considered in the analysis. ^b^ CSH/CSID did not propose specific ULN for ALT. The conventional values of 40 U/L for males and 40 U/L for females were adopted in the present analysis according to CSH/CSID 2019. ^c^ AASLD used ALT ULN levels of 35 U/L for males and 25 U/L for females.

**Table 2:** Characteristics of the grey zone patients as per the AASLD 2018 and the CSH/CSID 2022.

	AASLD 2018 N = 1971	CSH/CSID 2022 N = 1444	*p* Value
Age	42.0 (32.9, 51.6)	44.2 (35.0, 52.4)	0.001
% Male	1267 (64.3)	835 (57.8)	0.000
% HBeAg-positive	966 (49.0)	563 (39.0)	<0.0001
PLT (×10^9^/L)	187.0 (141.0, 228.0)	188.0 (145.0, 228.0)	0.778
ALT (U/L)	37.0 (25.0, 50.0)	26.0 (20.0, 35.0)	<0.0001
AST (U/L)	29.4 (23.4, 40.0)	25.0 (21.0, 33.0)	<0.0001
Alb (g/L)	44.0 (40.5, 46.6)	44.0 (40.8, 46.6)	0.844
TBil (μmol/L)	15.1 (11.4, 21.2)	14.6 (11.0, 20.4)	0.059
HBV DNA (log_10_ IU/mL)	3.9 (2.9, 5.2)	3.6 (2.0, 4.5)	<0.0001
APRI	0.4 (0.3, 0.7)	0.3 (0.3, 0.5)	<0.0001
% APRI ≥ 1.5	224 (11.4)	114 (7.9)	0.001
FIB-4, Median	1.1 (0.7, 2.1)	1.2 (0.8, 2.0)	0.218
% FIB-4 ≥ 3.25	278 (14.1)	188 (13.0)	0.389

Abbreviations: AASLD, American Association for the Study of Liver Diseases; Alb, albumin; ALT, alanine aminotransferase; APRI, AST-to-platelet ratio; AST, aspartate aminotransferase; CSH/CSID, Chinese Society of Hepatology/Chinese Society of Infectious Diseases; FIB-4, fibrosis score based on four factors; HBeAg, hepatitis B e antigen; HBV, hepatitis B virus; PLT, platelet; TBil, total bilirubin. Values are presented as number (%) for categorical variables or median (IQR) for continuous variables.

**Table 3:** Characteristics of grey zone patients as per the AASLD 2018 and CSH/CSID 2022: subgroup analysis by age group and sex.

	AASLD 2018 (N = 1971)						CSH/CSID 2022 (N = 1444)					
	Age < 30 N = 338 n (%)	Age ≥ 30 N = 1633 n (%)	*p* Value	Male N = 1267 n (%)	Female N = 704 n (%)	*p* Value	Age < 30 N = 199 n (%)	Age ≥ 30 N = 1245 n (%)	*p* Value	Male N = 835 n (%)	Female N = 609 n (%)	*p* Value
FIB-4 ≥ 3.25	10 (3.0)	268 (16.4)	<0.001	190 (15.0)	88 (12.5)	0.145	7 (3.5)	181 (14.5)	<0.001	125 (15.0)	63 (10.3)	0.012
APRI ≥ 1.5	25 (7.4)	199 (12.2)	0.015	167 (13.2)	57 (8.1)	0.001	11 (5.5)	103 (8.3)	0.233	81 (9.7)	33 (5.4)	0.004
ALT ≥ 40 U/L	178 (52.7)	697 (42.7)	0.001	717 (54.6)	158 (22.4)	<0.001	33 (16.6)	185 (14.9)	0.600	162 (19.4)	56 (9.2)	<0.001
PLT < 150 × 10^9^/L	65 (19.2)	516 (31.6)	<0.001	406 (32.0)	175 (24.9)	0.001	38 (19.1)	359 (28.8)	0.006	253 (30.3)	144 (23.7)	0.006

Abbreviations: AASLD, American Association for the Study of Liver Diseases; ALT, alanine aminotransferase; APRI, aspartate aminotransferase-to-platelet ratio; CSH/CSID, The Chinese Society of Hepatology and Chinese Society of Infectious Disease; FIB-4, fibrosis score based on four factors; PLT, platelet.

## Data Availability

All data analyzed in this study are included in this article and its supplementary material files. Further enquiries can be directed to the corresponding author.

## Supplementary Materials

The following supporting information can be downloaded at: https://www.mdpi.com/article/10.3390/v15051212/s1. Figure S1: Flowchart of patient selection; Table S1: Patient characteristics; Table S2: Characteristics of the inconsistent grey zone patients as per the AASLD 2018 alone and the CSH/CSID 2022 alone; Table S3: Distribution of clinical phases with varying ALT ULN values as per the AASLD 2018 and the CSH/CSID 2022.
