# Supplementary material for: The Impact of the Definitions of Clinical Phases on the Profiles of Grey-Zone Patients with Chronic Hepatitis B Virus Infection
Source: Viruses. 2023 May 22;15(5):1212. doi: 10.3390/v15051212 (PMC10222301; doi:10.3390/v15051212)
Supplement: Supplementary file 1 [file viruses-15-01212-s001.zip › viruses-2359250-supplementary.pdf]

Supplementary Figure S1 Flowchart of patient selection

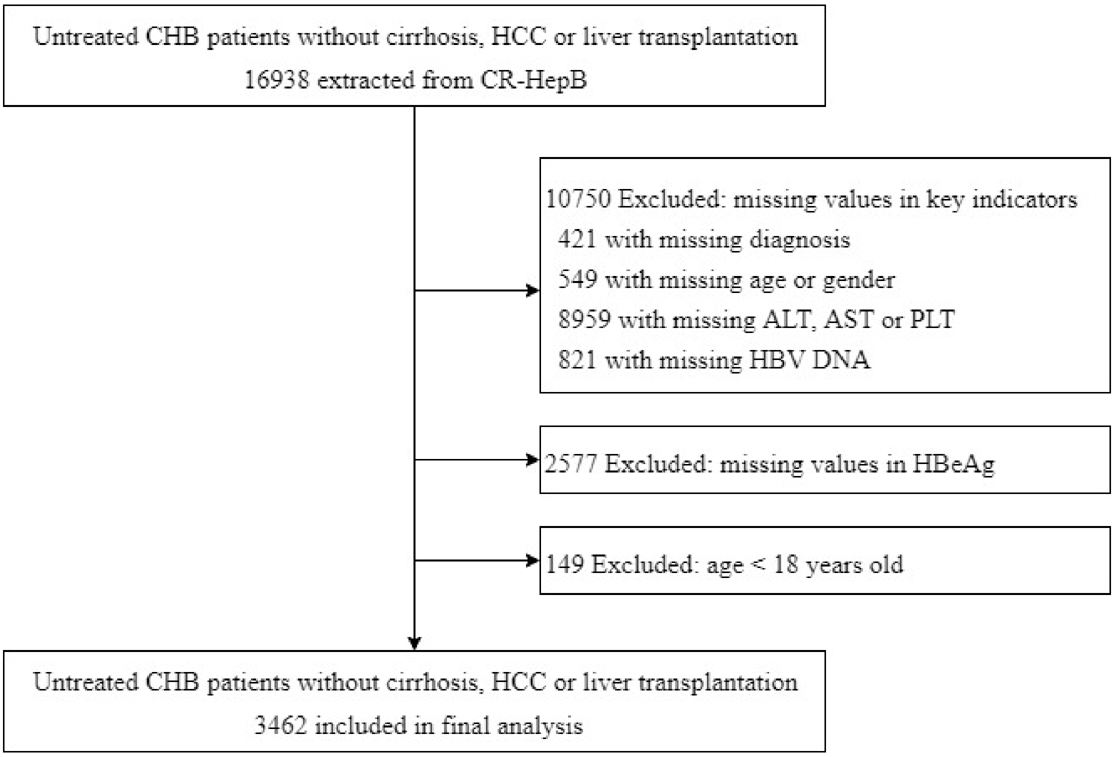

ALT, alanine aminotransferase; AST, aspartate aminotransferase; HBeAg, hepatitis B e antigen; HBV, hepatitis B virus; HCC, hepatocellular carcinoma; PLT, platelet

Supplementary Table S1 Patient characteristics

	**Total population**
Age	40.6 (31.3, 50.5)
% Male	2224 (64.2)
% HBeAg-positive	1802 (52.1)
PLT (×10^9/L)	188.0 (143.0, 229.0)
ALT (U/L)	39.0 (24.0, 78.0)
AST (U/L)	31.0 (23.0, 56.0)
Alb (g/L)	43.7 (40.0, 46.3)
TBil (μmol/L)	15.1 (11.4, 22.0)
HBV DNA (log_{10} IU/mL)	4.5 (3.0, 6.8)
APRI	0.4 (0.3, 1.0)
% APRI ≥ 1.5	592 (17.1)
FIB-4, Median	1.2 (0.7, 2.1)
% FIB-4 ≥ 3.25	523 (15.1)

Abbreviations: ALT, alanine aminotransferase; APRI, AST to platelet ratio; AST, aspartate aminotransferase; FIB-4, fibrosis score based on four factors; HBeAg, hepatitis B e antigen; HBsAg, hepatitis B surface antigen; HBV, hepatitis B virus; PLT, platelet; TBil, total bilirubin.

Values are presented as number (%) for categoric variables or median (IQR) for continuous variables.

Supplementary Table S2 Characteristics of the inconsistent grey zone[a] patients as per the AASLD 2018 alone and the CSH/CSID 2022 alone

	**Inconsistent grey-zone patients**		
	As per the AASLD 2018 alone N=777	As per the CSH/CSID 2022 alone N=250	*p* Value
Age	38.2 (30.2, 49.3)	42.2 (33.2, 49.3)	0.010
% Male	599 (77.1)	167 (66.8)	0.002
% HBeAg positive	485 (62.4)	82 (32.8)	<0.001
PLT ($\times 10^9$/L)	187.0 (138.0, 229.0)	191.0 (150.0, 229.8)	0.372
ALT (U/L)	48.0 (42.0, 60.0)	21.0 (16.0, 26.0)	<0.001
AST (U/L)	34.0 (28.0, 46.0)	21.6 (18.2, 26.0)	<0.001
Alb (g/L)	44.3 (40.9, 46.9)	45 (42.1, 47.3)	0.015
TBil (μmol/L)	15.46 (11.7, 21.7)	13.5 (9.7, 18.5)	<0.001
HBV DNA ($\log_{10}$ IU/mL)	4.9 (3.6, 7.4)	3.0 (2.5, 6.4)	<0.001
APRI	0.5 (0.3, 0.8)	0.3 (0.2, 0.4)	<0.001
% APRI ≥ 1.5	112 (14.4)	2 (0.8)	<0.001
FIB-4	1.1 (0.6, 2.0)	1.0 (0.7, 1.6)	0.931
% FIB-4 ≥ 3.25	107 (13.8)	17 (6.8)	0.005

Abbreviations: AASLD, American Association for the Study of Liver Diseases; ALT, alanine aminotransferase; APRI, AST to platelet ratio; AST, aspartate aminotransferase; CSH/CSID, The Chinese Society of Hepatology and Chinese Society of Infectious Disease; FIB-4, fibrosis score based on four factors; HBeAg, hepatitis B e antigen; HBsAg, hepatitis B surface antigen; HBV, hepatitis B virus; PLT, platelet; Tbil, total bilirubin.

Values are presented as number (%) for categoric variables or median (IQR) for continuous variables.

[a] Inconsistent grey-zone patients referred to the grey-zone patients excluding those consistently defined as grey-zone by both guidelines.

Supplementary Table S3 Distribution of clinical phases with varying ALT ULN values as per the AASLD 2018 and the CSH/CSID 2022

	Phase 1 n (%)	Phase 2 n (%)	Phase 3 n (%)	Phase 4 n (%)	Grey Zone n (%)
AASLD 2018					
ALT 50/40 U/L	456 (13.2)	393 (11.4)	514 (14.9)	172 (5.0)	1927 (55.7)
ALT 40/40 U/L	384 (11.1)	452 (13.1)	463 (13.4)	219 (6.3)	1944 (56.2)
ALT 35/25 U/L	274 (7.9)	562 (16.2)	386 (11.2)	269 (7.8)	1971 (56.9)
ALT 30/19 U/L	195 (5.6)	671 (19.4)	301 (8.7)	322 (9.3)	1973 (57.0)
CSH/CSID 2022					
ALT 50/40 U/L	312 (9.0)	833 (24.1)	287 (8.3)	415 (12.0)	1615 (46.7)
ALT 40/40 U/L	273 (7.9)	966 (27.9)	257 (7.4)	522 (15.1)	1444 (41.7)
ALT 35/25 U/L	192 (5.6)	1156 (33.4)	218 (6.3)	708 (20.5)	1188 (34.3)
ALT 30/19 U/L	135 (3.9)	1280 (37.0)	171 (4.9)	880 (25.4)	996 (28.8)

Abbreviations: AASLD, American Association for the Study of Liver Diseases; ALT, alanine aminotransferase; CSH/CSID, The Chinese Society of Hepatology and Chinese Society of Infectious Disease
